# Supplementary material for: Evolutionary Consequences of Repeated Loss of Distyly in Linum
Source: Genome Biol Evol. 2026 Sep 24;18(10):evag234. doi: 10.1093/gbe/evag234 (PMC13669628; doi:10.1093/gbe/evag234)
Supplement: Supplementary file 1 [file evag234_supplementary_data.pdf]

Evolutionary consequences of repeated loss of distyly in *Linum* spp.

Supplementary material

Supplementary Figures

Figure S1 – Example of a reconstructed chloroplast genome assembly

Figure S2 – ASTRAL phylogeny based on the 217 gene trees

Figure S3 – Geographical distribution of *Linum leonii* and *Linum perenne*

Figure S4 – PCA plots with both species' populations

Figure S5 – Demographic history 2D models

Supplementary tables

Supplementary table S1 – Sample information per species

Supplementary table S2 – GetOrganelle and GeSeq annotation metrics for the chloroplast genome assembly of each species

Supplementary table S3 – Hypothesis tested with PAML-codeml, Branch-model

Supplementary table S4 – Log likelihood-ratio test (LRT) results for D1 nuclear genes

Supplementary table S5 – d_N/d_S values for D1 nuclear genes, estimated using PAML-codeml, Branch-model H2

Supplementary table S6 – *Linum leonii* genome assembly metrics

Supplementary table S7 – *Linum leonii* genome assembly transposable elements (TEs) composition.

Supplementary table S8 – Sample information for *Linum leonii* and *Linum perenne* population genomic analyses

Supplementary table S9 – Read mapping information

Supplementary table S10 – ADMIXTURE cross-validation values

Supplementary table S11 – *dadi* 2D models analysis parameters

Supplementary table S12 – Hybpiper statistics

Supplementary dataset

Supplementary dataset S1 – Genome assembly report for *Linum leonii*

Supplementary Notes

Supplementary Note S1 - Demographic history inference for 2D SFS between *L. leonii* and populations of *L. perenne* ger3 and ger5.

Supplementary Note S2 – fastDFE best supported models

SUPPLEMENTARY FIGURES

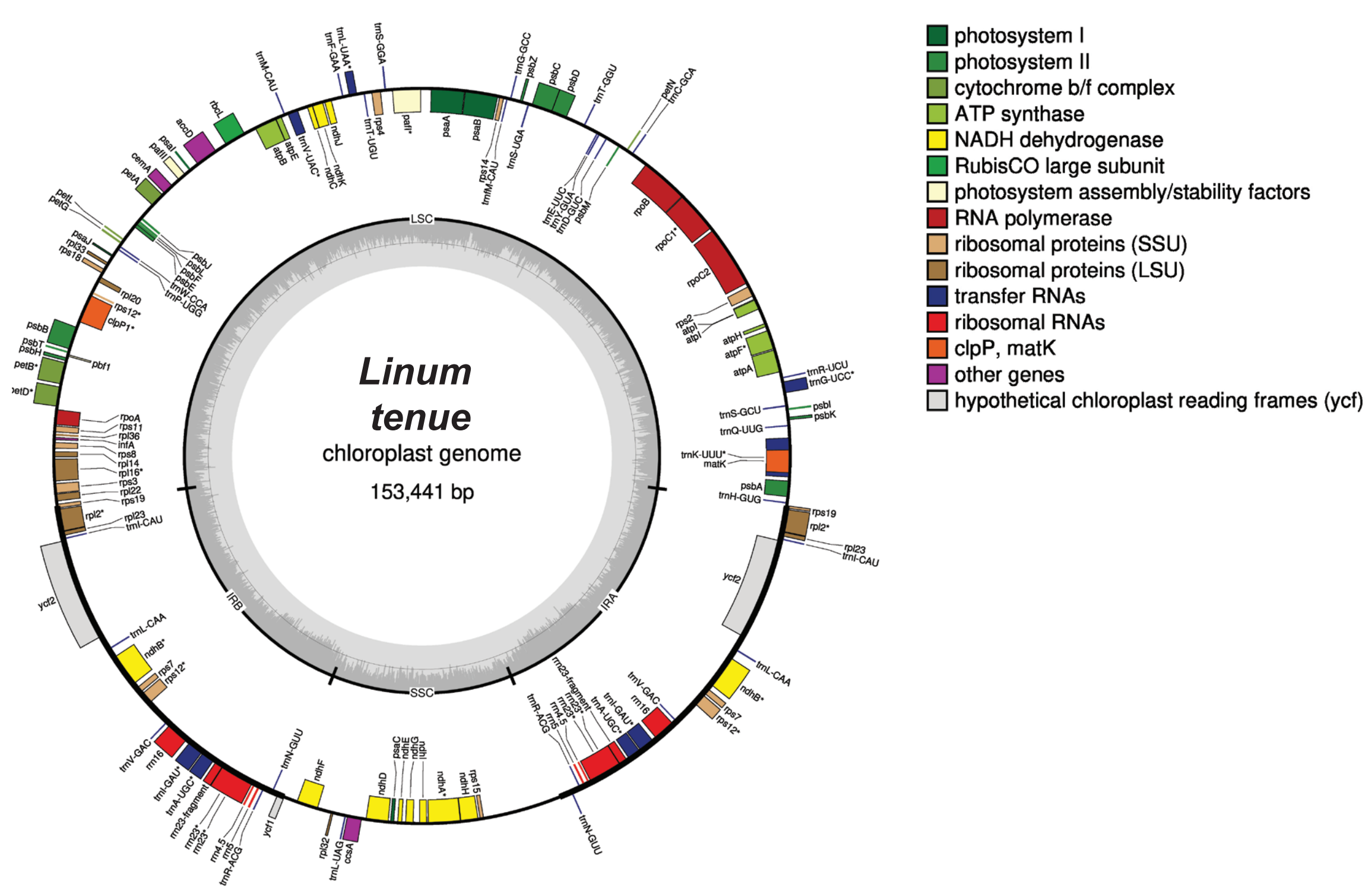

Figure S1 – Example of a reconstructed chloroplast genome assembly. The GC content is displayed on first inner circle. The limits of the Small and Large Single Copy Region (SSC and LSC) as well as limits of the inverted repeat (IR) can be found on the second inner circle. Gene's length and position can be retrieved on the chloroplast genome maps third circle. Each gene is colored depending on its chloroplast complex. The chloroplast genome size is indicated at the center of the map. Sizes of the different parts of the chloroplast genome (i.e. LSC, SSC, IR) can be found in Suppl. Table S2.

Alt. Text: Examples of one of the reconstructed chloroplast genome. The exact length of the IR, SSC and LSC can be found in Suppl. Table S2.

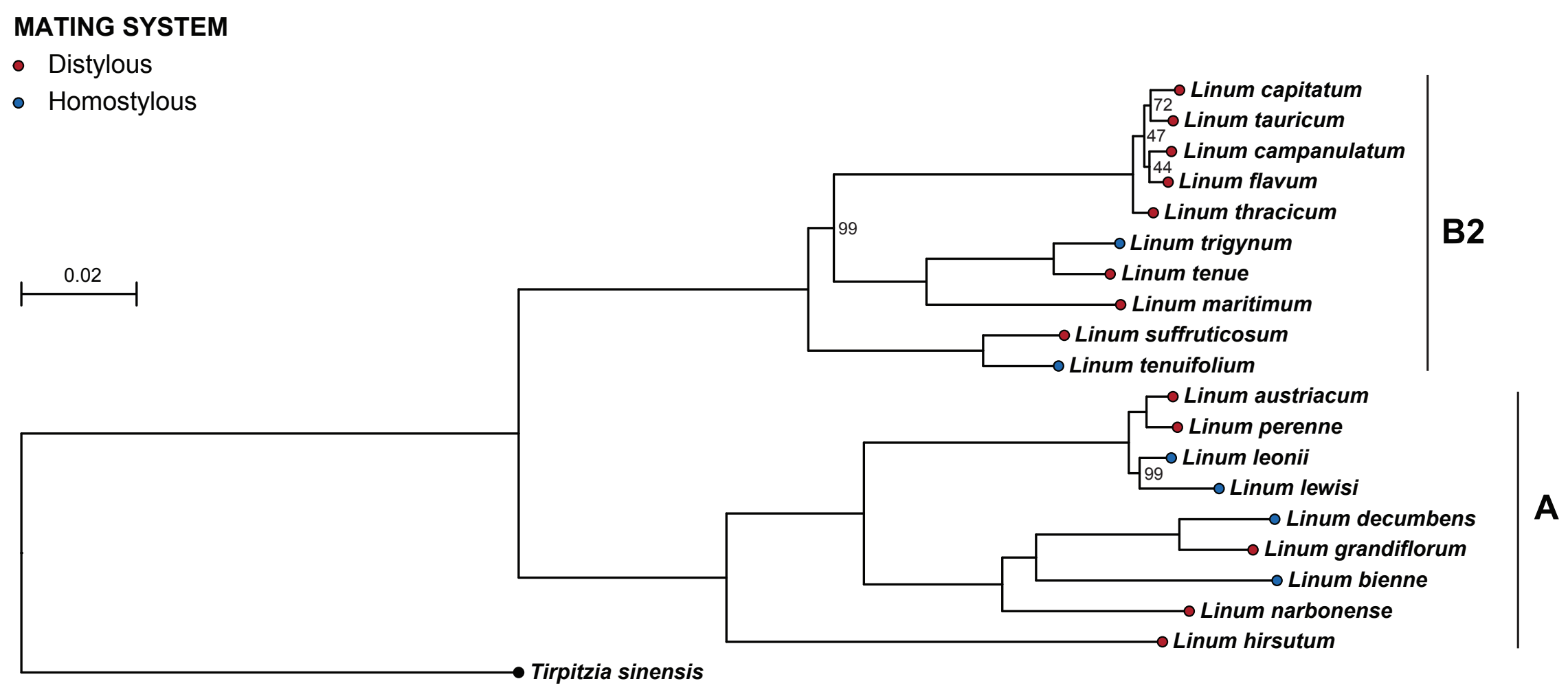

Figure S2 - ASTRAL phylogeny based on the 129 gene trees. This ASTRAL phylogeny was built using the 129 gene tree obtained with RAxML. Branch length and node were obtained running RAxML with a GTR GAMMA model of nucleotide substitution and a rapid bootstrap analysis with 1000 replicates on the tree built with the concatenation of the 129 genes (i.e. D2 dataset). Only nodes support with bootstrap value <100 are displayed. Species phylogenetic relationships are the same than the one observed with the chloroplast phylogeny. The only difference is the positioning of L. thracicum in clade B2 but the node support is quite low suggesting that this node is not well resolved as for the chloroplast data.

Alt. Text: ASTRAL phylogeny built with the 129 nuclear gene trees. Node supports with value <100 are displayed.

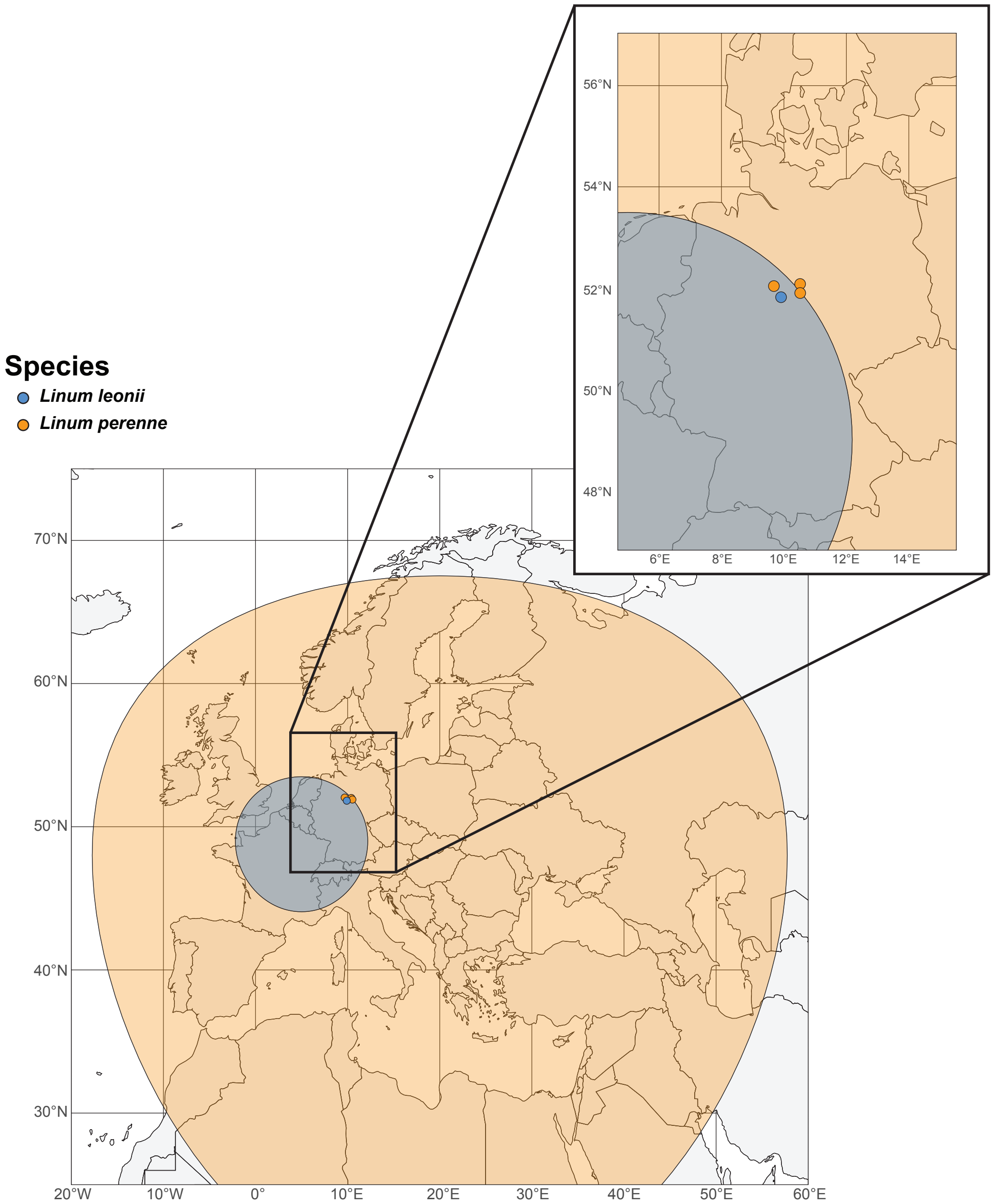

Figure S3 – Geographical distribution of *Linum leonii* and *Linum perenne*. *L. leonii* is represented in blue and *L. perenne* in orange. Sampled populations for both species are displayed and their geographical location zoomed in in the upper panel. Data were collected from GBIF and refined based on previous publications (Ockendon 1968, McDill et al. 2009, Maguilla et al. 2021). The figure was built on R.

Alt. Text*:* Geographical distribution of *Linum leonii* and *Linum perenne* and sampled populations for both.

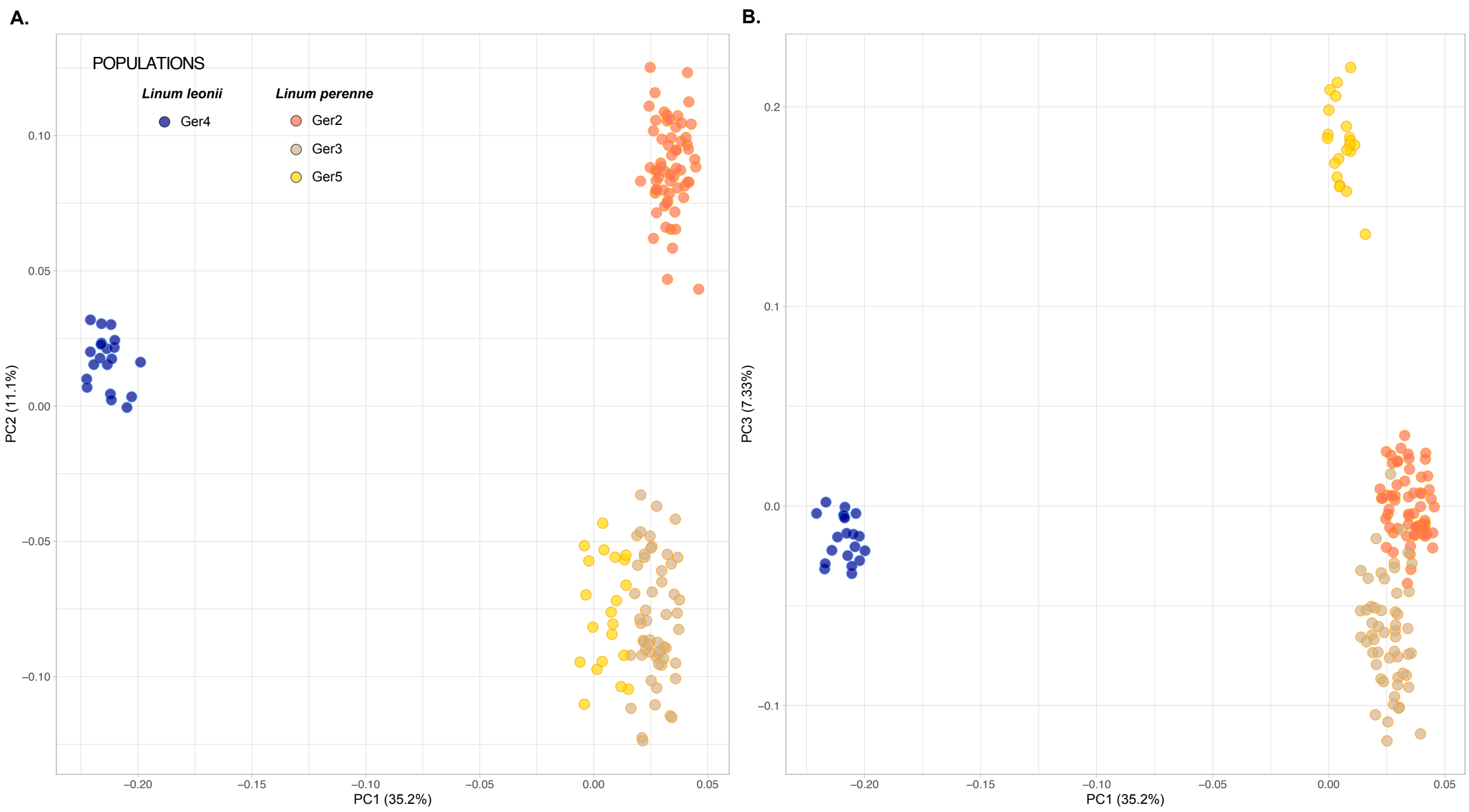

Figure S4 – Principal Component Analysis (PCA) plot with both species' populations: (A) PC1 and PC2; (B) PC1 and PC3. Individuals correctly clustered within each population. Additionally, both species separated along PC1 and PC2. *L. perenne*'s population ger2 differentiate from the other ones along PC2 and population ger5 differentiate along PC3 suggesting that population ger3 might be slightly admixed with both ger2 and ger5.

Alt. Text: Principal Component Analysis plot exhibiting the correct clustering of *L. perenne* and *L. leonii* populations.

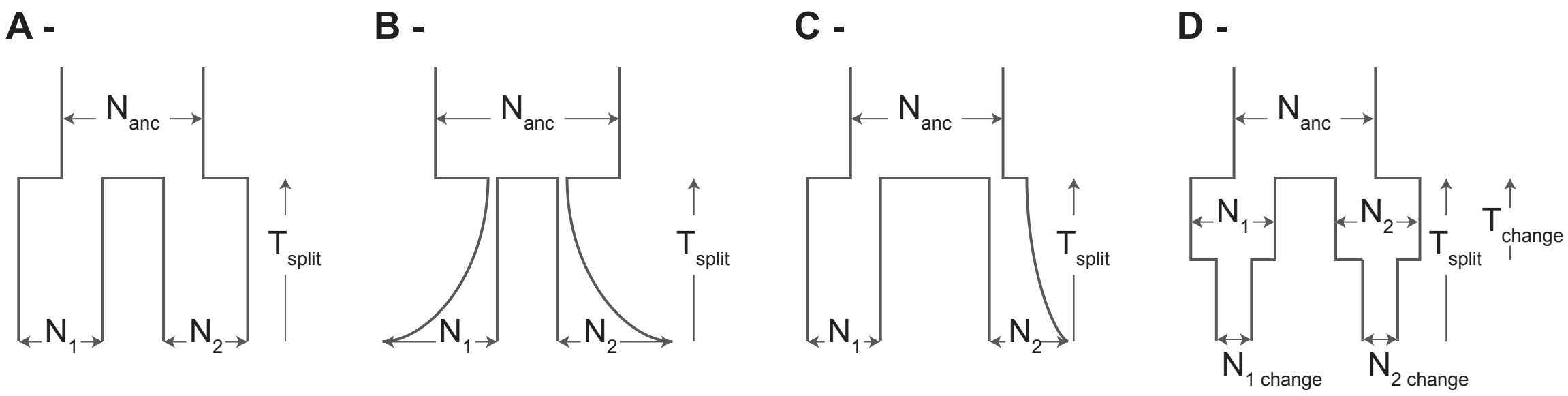

Figure S5 - Demographic history 2D models. Schematic representation of the tested demographic history models for joint site frequency spectra. The exponential models (C, D) can represent gradual increase or decrease in effective population size. Abbreviations are as described in Suppl. Table S11. The models' descriptions are the following:

- *split_no_growth* **(A)**: Split at T_split; Immediately two constant-size populations (N1, N2) without growth; Inbreeding (F_1, F_2).
- *vic_nomig* **(A)**: Split at T_split with proportions s and (1-s); Immediately two constant-size populations (N1, N2) without growth; Inbreeding (F_1, F_2).
- *split_growth* **(B)**: Split at T_split; Two exponential growing populations; Inbreeding (F_1, F_2).
- *split_growth_s* **(B)**: Split at T_split with proportions s and (1-s); Two exponentially growing populations; Inbreeding (F_1, F_2).
- *Founder_nomig* **(C)**: Split at T_split; pop2 starts at fraction s of ancestral Ne, pop1 at 1–s; Pop2 grows exponentially to N2; pop1 stays constant at N1; Inbreeding (F_1, F_2).
- *no_mig_size* **(D)**: Split at T_split; after split, each daughter population has constant size (N1, N2); Then at time T_change, both change to sizes (N1_change, N2_change); Inbreeding (F_1, F_2).

Alt. Text: The different demographic history models tested with dadi. Details about the parameters of these models can be found in Suppl. Table S11.

SUPPLEMENTARY TABLES

Legends

Supplementary table S1 – Sample information per species. Sample information for all *Linum* species used to estimates selective pressure in the homostylous vs distylous species. This tables report all information such as the source of the sample, the NCBI reference for chloroplast and nuclear data for the species that were already publicly available, the taxonomic section of the sample, its mating system and life style.

Supplementary table S2 – GetOrganelle and GeSeq annotation metrics for the chloroplast genome assembly of each species. Plastome assemblies' metrics and annotation for all twenty species. This table reports k-mer and assembly coverage as well as the number of mapped reads and whether GetOrganelle managed to retrieve the circular structure of the plastome assembly. We are also reporting the size of the plastome assembly as well as the size of its different parts (long and small single copy and the inverted repeat).

Supplementary table S3 – Hypothesis tested with PAML-codeml, Branch-model. Explanation of the different nested model ran on PAML – codeml. H0 correspond to the unannotated phylogenetic tree, thus with only one estimation of the d_N/d_S ratio for the whole tree. H1 correspond to the phylogenetic tree annotated with three different groups: distylous / homostylous / background, so with three independent estimations of the d_N/d_S ratio (i.e. one per group). H2 corresponds to the annotation of the phylogenetic tree into five different groups: clade A – distylous / clade A – homostylous / clade B2 – distylous / clade B2 - homostylous / background, thus with five different estimations of the d_N/d_S ratio.

Supplementary table S4 – Log likelihood-ratio test (LRT) results for D1 nuclear genes. Results of the log-likelihood ratio test for the nuclear D1 dataset. The LRT were done between H0 vs H1 and then H1 vs H2. As for the D2 dataset, the best branch model was H2 (i.e. one d_N/d_S ratio estimated per clade and per mating-system).

Supplementary table S5 – d_N/d_S values for D1 nuclear genes, estimated using PAML-codeml, Branch-model H2. Estimated d_N/d_S ratio based on the H2 branch model, for the D1 nuclear genes set. As for the D2 dataset, clade A homostylous species exhibit slightly elevated d_N/d_S ratio compared to the distylous species while no differences are observed between homostylous and distylous species of clade B2.

Supplementary table S6 – *Linum leonii* genome assembly statistics. Report of the main metrics for the newly built genome assembly of *L. leonii*. The assembly of length 889 Mb is of good quality, with a BUSCO score of 93.9%.

Supplementary table S7 – *Linum leonii* genome assembly transposable elements (TEs) composition.

Supplementary table S8 – Samples information for *Linum leonii* and *Linum perenne* population's genomic analyses. This table reports information of the sampling location of the samples used to test for the genomic selfing in syndrome in the homostylous *L. leonii* in contrast to its distylous relatives, *L. perenne*. It also reports the number of samples for each population.

Supplementary table S9 – Read mapping information to get the different VCF files. This tables details which reads were mapped to which assembly for each analysis. The VCF files were then built based on these mapped reads.

Supplementary table S10 – ADMIXTURE cross-validation values. Reports of the different CV values for each cluster number (K). The lowest CV value was retrieved for K8 indicating that *L. leonii* and *L. perenne* individuals' group into eight different genetic cluster.

Supplementary table S11 – *dadi* 2D best-model parameters. Report of the different parameters for dadi 2D best-fitting models for comparison between *L. leonii* and each population of *L. perenne*. Schematic representations of the models are shown in Suppl. Fig. S5.

Supplementary table S12 – Hybpiper statistics. This table reports the main statistics from Hybpiper pipeline, such as the total number of reads and the number of mapped reads and the number of genes with a paralog warning, per species.

Suppl. Table S1 – Sample information per species.

Species	Source	Chloroplast data	Nuclear data	Section*	Mating system	Clade	Life style**
Linum hirsutum	LIN 2100 (IPK)	Short read, WGS	Target Capture	Dasylinum	Distylous	Clade A	Perennial
Linum austriacum	LIN 1883 (IPK)	Short read, WGS	Target Capture	Linum	Distylous	Clade A	Perennial
Linum grandiflorum	LIN 974 (IPK)	Short read, WGS	Short read, WGS	Linum	Distylous	Clade A	Annual
Linum narbonense	LIN 1807 (IPK)	Short read, WGS	Target Capture	Linum	Distylous	Clade A	Perennial
Linum perenne	LIN 2003 (IPK)	Short read, WGS	Target Capture	Linum	Distylous	Clade A	Perennial
Linum bienne	-	SRR1592607	SRR1592607	Linum	Homostylous	Clade A	Annual
Linum decumbens	-	MV366795.1	SRR1592610	Linum	Homostylous	Clade A	Annual
Linum leonii	18-2, ger2, 2018 near Moringen, Germany	Short read, WGS	Target Capture	Linum	Homostylous	Clade A	Perennial
Linum lewisii	-	NC_058799.1	SRR1592654	Linum	Homostylous	Clade A	Perennial
Linum thracicum	LIN 1553 (IPK)	Short read, WGS	Target Capture	-	Distylous	Clade B2	Perennial
Linum maritimum	MAR-12, Soler botanical garden, Spain	Short read, WGS	Target Capture	Linopsis B	Distylous	Clade B2	Perennial
Linum tenue	Lten-21-22, SAMEA114529652	Short read, WGS	Target Capture	Linopsis B	Distylous	Clade B2	Annual
Linum trigynum	Ltri-10-7, SAMEA114529727	Short read, WGS	Target Capture	Linopsis B	Homostylous	Clade B2	Annual
Linum suffruticosum	18-5-2 near Ronda, Spain	Short read, WGS	Target Capture	Linopsis C	Distylous	Clade B2	Perennial
Linum tenuifolium	LIN 1759 (IPK)	Short read, WGS	Target Capture	Linopsis C	Homostylous	Clade B2	Perennial
Linum campanulatum	LIN 1760 (IPK)	Short read, WGS	Target Capture	Syllinum	Distylous	Clade B2	Perennial
Linum capitatum	LIN 1903 (IPK)	Short read, WGS	Target Capture	Syllinum	Distylous	Clade B2	Perennial
Linum flavum	LIN 2001 (IPK)	Short read, WGS	Target Capture	Syllinum	Distylous	Clade B2	Perennial
Linum tauricum	LIN 1658 (IPK)	Short read, WGS	Target Capture	Syllinum	Distylous	Clade B2	Perennial
Tirpitzia sinensis	-	ON881454.1	ERR9230207	-	Distylous	Outgroup	Na

*Maguilla et al. 2021
**References for mating system and life habits: Ruiz-Martin et al. 2018, Maguilla et al. 2021, Valdes-Florido et al. 2023, Tork et al. 2021, Jahnke and Etterson. 2018, Link

Suppl. Table S2 – GetOrganelle and GeSeq annotation metrics for the chloroplast genome assembly of each species.

**Clade**	**Mating system**	**Species**	**Kmer cov**	**Asm cov**	**Nb mapped reads**	**Graph (K115)**	**Size – in bp**			
							**Total asm**	**LSC**	**IR**	**SSC**
Clade A	Distylous	*L. austricanum*	116.4	475.0x	271,381	Circular	172,330	72,208	44,567	10,988
		L. grandiflorum	130.7	533.3x	283,580	Circular	158,174	82,760	32,480	10,454
		L. hirsutum	123.5	504.1x	276,026	Circular	165,215	79,881	38,237	8,860
		L. narbonense	124.3	507.2x	272,110	Circular	159,233	83,040	32,665	10,863
		L. perenne	117.2	478.1x	273,028	Circular	172,281	72,207	44,543	10,988
	Homostylous	*L. bienne*	73.9	462.0x	385,165	Circular*	153,096	81,759	32,117	10,951
		L. decumbens	-	-	-	-	157,739	80,618	32,321	10,458
		L. leonii	120.5	492x	282,740	Circular	173,198	72,468	44,895	10,940
		L. lewisii	-	-	-	-	159,778	83,267	32,810	10,891
Clade B2	Distylous	*L. campanulatum*	123.9	505.8x	259,104	Circular	153,941	83,701	25,768	18,704
		L. capitatum	122.8	501.2x	255,813	Circular	153,941	83,701	25,768	18,704
		L. flavum	122.4	499.4x	252,293	Circular	153,940	83,698	25,768	18,706
		L. maritimum	115.4	471.0x	241,529	Circular	153,917	83,543	25,642	19,090
		L. suffricatosum	107.5	438.8x	226,675	Circular	153,455	83,514	25,592	18,757
		L. tauricum	121.8	496.9x	254,138	Circular	153,828	83,586	25,768	18,706
		L. tenue	119.3	486.7x	249,133	Circular	153,441	83,393	25,646	18,756
		L. thracicum	113.1	461.6x	236,761	Circular	153,941	83,701	25,768	18,704
	Homostylous	*L. teniufolium*	125.5	512.1x	261,892	Circular	153,393	83,499	25,578	18,738
		L. trigynum	122.4	499.4x	255,131	Circular	153,257	83,147	25,603	18,904
Outgroup	Distylous	*T. sinensis*	-	-	-	-	154,702	84,141	25,827	18,907
Mean			117.51	490.45	264,160	-	157,405	81,612	29,659	14,796

Cov: coverage; **Asm**: assembly; **Graph**: shape of the graph produced by both GetOrganelle and GeSeq; **LSC**: Large Single Copy region; **IR**: Inverted Repeat; **SSC**: Small Single Copy region; **K115**: the size of the k-mer used to construct the assembly and the graph; **"*":** for this species, the k-mer size was of 85 and not 115; **"-":** data from NCBI so the assembly was already available and we just conducted annotation for these species.

Suppl. Table S3 – Hypotheses tested using PAML-codeml, Branch Model

**H0**	One dN/dS for the whole tree
**H1**	One dN/dS per mating-system
**H2**	One dN/dS per mating-system and per clade

Suppl. Table S4 – Log likelihood-ratio test results for D1 nuclear genes

**D1, nuclear tree**	**LRT**	**DDL**	**PVALUES**
Whole VS mating-system	30.90	2	<0.001
Mating-system VS Clade + mating-system	6.31	2	0.04

Suppl. Table S5 – dN/dS values for nuclear datasets D1 estimated using PAML – codeml, H2 branch-model

	**CLADE A**	**CLADE B2**
**Distylous**	0.10 ± 0.003	0.11 ± 0.003
**Homostylous**	0.14 ± 0.008	0.12 ± 0.01

Suppl. Table S6 – Genome assembly statistics

**Metric**	**hifiasm+hic primary**
Number of contigs	850
Largest contig	54,108,257
Total length*	888,529,764
GC (%)	41.12
N50**	21,077,493
N75	9,821,036
L50***	12
L75	28
# N's per 100 kbp	0
BUSCO	93.9%

*Flow-cytometry based genome size estimates for *L. leonii* is 983 Mb
**N50: 50% of contigs in the assembly are longer than the N50 length; N75: 75%
***L50: Count of the smallest number of contigs which together make up 50% of the genome assembly size; L75: same but up 75%

Suppl. Table S7 - TE composition of the *L. leonii* genome assembly.

Repeats Category	Number of elements	Length (bp)	Percentage of sequence
**Retroelements**	490,427	520,484,188	58.6
SINEs	2,069	316,608	0.04
LINEs	31,152	28,943,163	3.26
RTE/Bov-B	591	58,976	0.01
L1/CIN4	30,348	28,840,452	3.25
LTR elements	457,206	491,224,417	55.3
Ty1/Copia	82,672	40,299,602	4.54
Ty3/DIRS1	372,632	450,485,038	50.72
Retroviral	1,902	439,777	0.05
**DNA transposons**	264,662	99,233,497	11.17
hobo-Activator	47,199	12,779,374	1.44
Tc1-IS630-Pogo	26,786	6,103,395	0.69
Tourist/Harbinger	35,897	10,476,496	1.18
Other (Mirage/P-element/Transib)	113	22,172	0
**Rolling-circles**	46,008	19,847,558	2.23
**Unclassified**	18,877	10,154,143	1.14
**Total interspersed repeats**	-	629,871,828	70.91
**Small RNA**	2,069	316,608	0.04
**Simple repeats**	73,815	3,199,627	0.36
**Low complexity**	11,88	599,02	0.07

Total length = 888,247,830 bp ; GC level = 41.12%

Suppl. Table S8 – Sample information for *L. leonii* and *L. perenne* population genomic analyses

Species	accession/origin	Lat	Lon	Nb of inds analyzed	Nb of downsampled inds
Linum perenne	ger2 near Othfresen, Germany	52,01	10,40	63	37
	ger3 near Marienhagen, Germany	52,04	9,68	54	33
	ger5 near Goslar, Germany	51,90	10,52	20	13
Linum leonii	ger4 near Einbeck, Germany	51,82	9,91	20	10
Linum leonii	near Moringen, Germany	51,73	9,80	1 (assembly)	
Linum grandiflorum	*Lgra-62-06-thrum; LIN 10 (IPK)*	NA	NA	1	

Suppl. Table S9 – Read mapping information

Analysis	Species' reads	Species' assembly for read mapping
**Inbreeding coefficient**	*L. leonii*	*L. leonii*
	L. perenne	*L. perenne*
**Genome-wide π**	*L. leonii*	*L. leonii*
	L. perenne	*L. perenne*
**Tajima's D**	*L. leonii*	*L. leonii*
	L. perenne	*L. perenne*
**piN/piS**	*L. leonii*	*L. leonii*
	L. perenne	*L. perenne*
**PCA**	*L. leonii* + *L. perenne*	*L. perenne*
**Admixture**	*L. leonii* + *L. perenne*	*L. perenne*
**Dadi**	*L. leonii* + *L. perenne*	*L. perenne*
**DFE**	*L. leonii* + *L. grandiflorum*	*L. leonii*
	L. perenne + *L. grandiflorum*	*L. perenne*

Suppl. Table S10 – ADMIXTURE Cross-validation values

K	CV value
K1	0.55028
K2	0.43650
K3	0.37169
K4	0.34807
K5	0.33965
K6	0.33442
K7	0.32908
K8	0.32303
K9	0.32337
K10	0.32648

Supplementary table S11 – *dadi* 2D model parameters and descriptions.

Pop. 1	Pop. 2	Best inferred model	Model parameters												Year-scaled parameters (generations * 2)	
			theta	L	Ne_ref	Tsplit	N1	N2	T change	N1 change	N2 change	F1	F2	AIC[1]	Tsplit scaled	Tchange scaled
L.per - ger2	Lleo	no_mig_size	2,715	66,186	1,444,645	897,528	4,328,838	3,036,232	62,356	33,871	11,352	0.01	0.20	4,348	1,795,056	124,713
Lper - ger3	Lleo	split_growth	3,186	66,116	1,696,929	496,799	115,634	18,129				0.00	0.55	4,57	993,598	
Lper - ger5	Lleo	split_growth	3,059	65,424	1,646,582	973,122	291,993	41,574				0.00	0.21	3,325	1,946,244	

Model description:
- *split_growth* (Suppl. Fig. S5-A): Split at Tsplit; two exponential growing populations; inbreeding (F_1, F_2).
- *no_mig_size* (Suppl. Fig. S5-D): Split at Tsplit; after split, each daughter population has constant size (N1, N2); then at time Tchange, both change to sizes (N1-change, N2-change); inbreeding (F_1, F_2).

[1]AIC scores are comparable among different tested scenarios for the same population pairs but not between population pairs. Only the score for best-supported demographic history model is presented in this table.

Suppl. Table S12 – Hybpiper statistics with number of mapped reads and genes with paralog warning for each sample.

	Species	Mating system	Number of reads from target capture		Number of gene with paralog warning	
			Total	Mapped	On gene's length	On reads depth
**Clade A**	*L. austriacum*	Distylous	20,228,044	6,972,245	8	9
	L. grandiflorum		216,368,754	178,416	11	21
	L. hirsutum		17,055,766	2,806,819	9	15
	L. narbonense		19,592,244	2,154,900	16	34
	L. perenne		16,814,040	3,028,139	15	21
	L. bienne	Homostylous	104,067,116	118,560	9	31
	L. decumbens		83,925,414	39,636	1	2
	L. leonii		18,208,918	5,236,548	11	18
	L. lewisii		84,213,206	49,799	2	3
**Clade B2**	*L. campanulatum*	Distylous	24,272,000	4,303,828	18	34
	L. capitatum		15,451,632	2,649,107	14	28
	L. flavum		15,987,986	2,723,333	17	32
	L. maritimum		17,128,434	1,861,584	42	78
	L. suffruticosum		16,395,940	4,249,703	19	73
	L. tauricum		14,253,776	2,421,890	14	29
	L. tenue		19,575,804	4,972,442	43	90
	L. thracicum		18,932,174	3,282,495	16	40
	L. tenuifolium	Homostylous	15,125,490	3,576,330	6	14
	L. trigynum		21,819,288	5,604,946	42	69

DATASET S1

BUSCO	
C:93.9%[S:88.7%,D:5.2%],F:1.3%,M:4.8%,n:2326	
2,184	Complete BUSCOs (C)
2,063	Complete and single-copy BUSCOs (S)
121	Complete and duplicated BUSCOs (D)
30	Fragmented BUSCOs (F)
112	Missing BUSCOs (M)
2,326	Total BUSCO groups searched

REPEAT MASKING						
**Species**	**Tool**	**Library**	**Number**	**Total size (kb)**	**Mean size (bp)**	**% Genome**
Linum leonii	Repeat masker	De-novo	722,584	665,784.20	921.39	74.95
Linum leonii	Repeat Runner	MAKER TE	30,091	26,258.26	872.63	2.96

INTERPROSCAN				
**Databases**	**Nb term linked to mRNA**	**Nb mRNA with term in raw file**	**Nb mRNA updated by term in our annotation file**	**Nb gene updated by term in our annotation file**
AntiFam	25	25	25	22
CDD	44,825	44,825	37,978	10,586
Coils	16,886	16,886	16,886	5,802
FunFam	62,387	62,387	38,625	10,034
GO	298,695	298,695	108,379	31,984
Gene3D	118,148	118,148	78,358	20,971
Hamap	3,098	3,098	3,032	1,184
InterPro	361,926	361,926	108,379	31,984
MetaCyc	21,552,435	21,552,435	79,135	21,527
MobiDBLite	46,057	46,057	46,057	17,801
PANTHER	106,857	106,857	106,857	31,456
PIRSF	4,824	4,824	4,760	1,479
PRINTS	19,772	19,772	17,049	4,181
Pfam	145,874	145,874	100,472	28,893
ProSitePatterns	27,351	27,351	21,710	5,275
ProSiteProfiles	51,123	51,123	39,918	11,783
Reactome	75,308,129	75,308,129	84,213	23,235

SFLD	2,787	2,787	1,374	328
SMART	40,428	40,428	32,187	8,232
SUPERFAMILY	97,098	97,098	76,603	20,527
TIGRFAM	10,595	10,595	97,75	2,852

**CODING GENES ANNOTATION STATISTICS**	
Description *Linum leonii*	
Number of protein-coding genes	45,638
Number of mRNA	13,2390
Average number of exons per mrna	5.3
Average exon length	299
Average intron length	342

SUPPLEMENTARY NOTES

<u>Supplementary Note S1:</u> Demographic history inference for 2D SFS between *L. leonii* and populations of *L. perenne* ger3 and ger5.

To estimate the divergence and subsequent demographic history of both species, we inferred split models between *L. leonii* and the three populations of *L. perenne* (ger2, ger3 and ger5) using *dadi* (Gutenkunst et al. 2009; Blischak et al. 2020) (Suppl. Fig. S5, Suppl. Table S10). Results for the split between *L. leonii* and *L. perenne* – ger2 are reported in the main text. Below you can find the results for the two other splits: *L. leonii* vs *L. perenne* - ger3 and *L. leonii* vs *L. perenne* - ger5. The best-fitting split model between *L. perenne* population ger3 and *L. leonii* was *"split_growth"* and indicated a split around 1 Mya. After divergence, both populations experienced exponential size reductions. Population *L. perenne* - ger3 has a final effective population size of ~115,000, while *L. leonii* stabilized at a much smaller effective size of ~18,000. Inbreeding was low in *L. perenne* - ger3 ($F_{ger3} < 0.01$) but higher in *L. leonii* ($F_{leonii\text{-}ger3} = 0.55$) (Suppl. Table S11). These results might be affected by a small amount of gene flow between these two populations which was inferred by ADMIXTURE (Alexander et al. 2009) and was not included in the demographic history models. The same model was preferred for the pair of *L. perenne* - ger5 and *L. leonii*. The split was inferred at ~2Mya and the final effective populations sizes were estimated at ~290,000 and ~40,000 for *L. perenne* - ger5 and *L. leonii* respectively. Inbreeding was similar to the *L. perenne* - ger2 vs *L. leonii* pair ($F_{ger5} < 0.01$, $F_{leonii\text{-}ger5} = 0.21$) (Suppl. Table S10).

Supplementary Note S2: **fastDFE best supported models**

The Distribution of Fitness Effect (DFE) was inferred using fastDFE (Sendrowski and Bataillon 2024) for each population. This software uses and transforms the neutral site frequency spectrum (SFS) to correct for the effect of demography and applies the same transformation to the selected SFS. The selected SFS is then compared to expectations under different distributions of fitness effects to estimate the parameters that best explain the data. The best supported models inferred combined gamma (for deleterious mutations) and exponential (for beneficial mutations) distributions (GammaExpParametrization()) for the three *L. perenne* populations' DFEs, and gamma deleterious-only distribution (using GammaExpParametrization() or GammaDiscreteParametrization()) for *L. leonii*.

References

Alexander DH, Novembre J, Lange K. 2009. Fast model-based estimation of ancestry in unrelated individuals. Genome Res. 19(9):1655–1664. https://doi.org/10.1101/gr.094052.109

Blischak PD, Barker MS, Gutenkunst RN. 2020. Inferring the Demographic History of Inbred Species from Genome-Wide SNP Frequency Data. Molecular Biology and Evolution. 37(7):2124–2136. https://doi.org/10.1093/molbev/msaa042

Gutenkunst RN, Hernandez RD, Williamson SH, Bustamante CD. 2009. Inferring the Joint Demographic History of Multiple Populations from Multidimensional SNP Frequency Data. PLOS Genetics. 5(10):e1000695. https://doi.org/10.1371/journal.pgen.1000695

Sendrowski J, Bataillon T. 2024. fastDFE: Fast and Flexible Inference of the Distribution of Fitness Effects. Molecular Biology and Evolution. 41(5):msae070. https://doi.org/10.1093/molbev/msae070
